# Magnetic resonance imaging appearance of hypertensive encephalopathy in a dog

**DOI:** 10.1186/s13620-015-0033-6

**Published:** 2015-04-24

**Authors:** Chloe A Bowman, Adrian Witham, Dayle Tyrrell, Sam N Long

**Affiliations:** Neurology Department, University of Melbourne Veterinary Clinic and Hospital, 250 Princes Highway, Werribee, Melbourne, 3030 Australia; Internal Medicine Department, University of Melbourne Veterinary Clinic and Hospital, 250 Princes Highway, Werribee, Melbourne, 3030 Australia; Diagnostic Imaging Department, University of Melbourne Veterinary Clinic and Hospital, 250 Princes Highway, Werribee, Melbourne, 3030 Australia

**Keywords:** Neurology, Canine, Magnetic resonance imaging, Diffusion weighted imaging

## Abstract

A 16-year-old female spayed English Staffordshire terrier was presented for evaluation of a 10-month history of intermittent myoclonic episodes, and a one weeks history of short episodes of altered mentation, ataxia and collapse. Magnetic resonance imaging identified subcortical oedema, predominately in the parietal and temporal lobes and multiple cerebral microbleeds.

Serum biochemistry, indirect blood pressure measurements and magnetic resonance imaging abnormalities were consistent with hypertensive encephalopathy secondary to chronic kidney disease.

## Background

Hypertension is a common sequel to chronic kidney disease (CKD), with the incidence of CKD associated systolic arterial hypertension (SAP) in dogs estimated at around 30% [1]. Neurological complications of hypertension that have been described in dogs and cats include cerebrovascular disease and hypertensive encephalopathy [2-4].

In humans, hypertensive encephalopathy is defined as a syndrome of neurological abnormalities including seizures, secondary to hypertension induced cerebral oedema [5,6]. This encephalopathy is now considered part of posterior reversible encephalopathy syndrome (PRES), a syndrome of clinical signs including headache, altered mentation and vision abnormalities. The main magnetic resonance imaging (MRI) abnormality is vasogenic oedema predominately affecting the occipital and parietal white matter [5-8]. Cerebral oedema secondary to hypertension induced PRES is reversible with anti-hypertensive therapy. A recent veterinary case series has described the MRI and clinicopathologic findings associated with hypertensive encephalopathy in 2 cats and 2 dogs [9]. These animals had MRI changes including occipital and parietal white matter oedema with concurrent systolic hypertension.

Cerebrovascular disease, and in particular intracerebral haemorrhage, is found in up to 33% of people with PRES [7]. Haemorrhage can be classified into 3 types; intraparenchymal haematoma, small haemorrhages <5 mm diameter and subarachnoid haemorrhage [5]. In people, the small regions of haemorrhage/haemolysis which have the imaging appearance of punctate signal voids on T2*-weighted gradient recalled-echo sequences (T2*-GRE) are termed cerebral microbleeds. These small, <5 mm diameter lesions, correspond histopathologically to areas of microhaemorrhage and/or products of haemolysis [10-13]. A veterinary case series has described the imaging characteristics of cerebral microbleeds in 4 dogs; 1 dog of which was found to be hypertensive [14].

## Case presentation

A 16-year old, female spayed, English Staffordshire terrier was presented to the University of Melbourne Veterinary Hospital for evaluation of episodes of myoclonic seizures of 10 months duration. Exposure to bright sunlight or quickly moving objects would result in generalized facial twitching, tonic muscular contractions mainly affecting the thoracic limbs, and an atonic phase with the dog falling backwards. The episodes had increased in frequency, and at the time of presentation, the dog exhibited these signs every few minutes when in full sunlight. Mentation between events was normal. Five days prior to presentation, there were further neurological signs that were triggered by a stressful event. This manifested as an episode of altered mentation with excitement and vocalization, and ataxia lasting a few minutes. This was followed by two short episodes of ataxia and collapse without loss of consciousness over the following week. The dog had a year history of progressive polyuria and polydipsia.

The University of Melbourne Veterinary Research Board approved the study.

Neurological examination was unremarkable aside from generalized facial twitching and tonic muscular contractions elicited by visual stimulation such as a hand moving quickly towards the dog. Fundic examination was normal. The dog had a known, complete vaccination history, and general physical examination was normal.

Results of a complete blood count, serum biochemistry profile and urinalysis revealed abnormalities consistent with chronic kidney disease. Serum urea nitrogen was moderately elevated (19.8 mmol/L; reference interval (RI): 3.5 to 11.1 mmol/L), as was serum creatinine (206 mmol/L; RI: 53 to 120 mmol/L). There was a mild normocytic normochromic non-regenerative anaemia: haematocrit 36% (RI: 37 to 55%). Urine specific gravity (USG) was 1.020. Systolic arterial blood pressure (SAP) averaged 170 mmHg by Doppler sphygmomanometry [reference limit (RL): <150 mmHg [2]]. Blood pressure readings were taken 6 hours after hospitalisation and the average of 5 readings over 20 minutes was recorded. Urine sediment was normal, as was urine protein-creatinine ratio 0f 0.15 (RL: <0.2). The dog was classified according to the scoring system of the Internal Renal Interest Society (IRIS 2009) as IRIS stage 3, non-proteinuric (NP), arterial pressure (AP) sub stage 2. Abdominal ultrasonography was unremarkable aside from a unilateral nephrolith with associated chronic renal parenchymal changes. Thoracic radiographs were not performed.

Magnetic resonance imaging of the brain was performed using a 1.5 T magnet (GE Healthcare: Sigma HD). T1-weighted (T1-W) 3D images were acquired pre and post contrast in the dorsal plane and reformatted in the transverse and sagittal planes. T2-weighted (T2-W) images were acquired in transverse and sagittal planes, fluid-attenuated inversion recovery (FLAIR) images were acquired in the transverse plane, T2*-weighted gradient recalled-echo (T2*-GRE) and diffusion-weighted images (DWI) were acquired in the transverse plane, and apparent diffusion coefficient (ADC) map in the transverse plane was calculated (GE software: Functool). The contrast agent gadopetetate dimeglumine (Magnevist^R^, Bayer Healthcare Pharmaceuticals) was administered intravenously at a dose of 0.1 mmol/kg.

**Figure 1:**
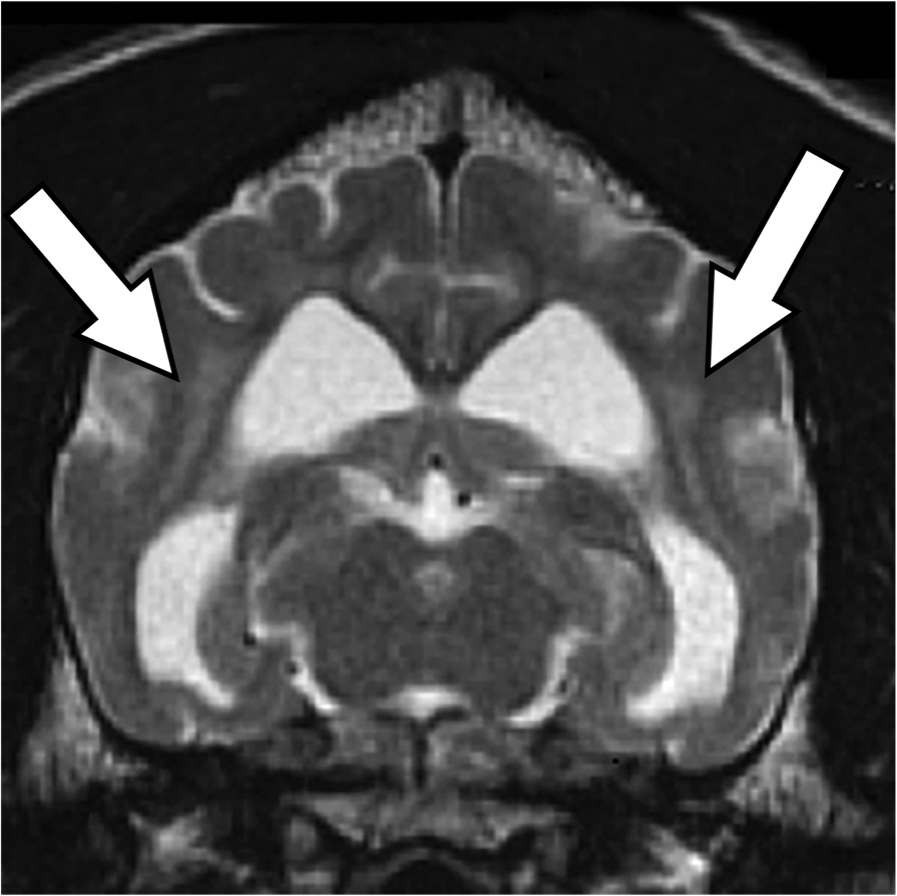
T2-W sequence at the level of the medial geniculate, showing a hyperintense signal in the internal capsule peripheral to the lateral ventricles (arrows).

**Figure 2:**
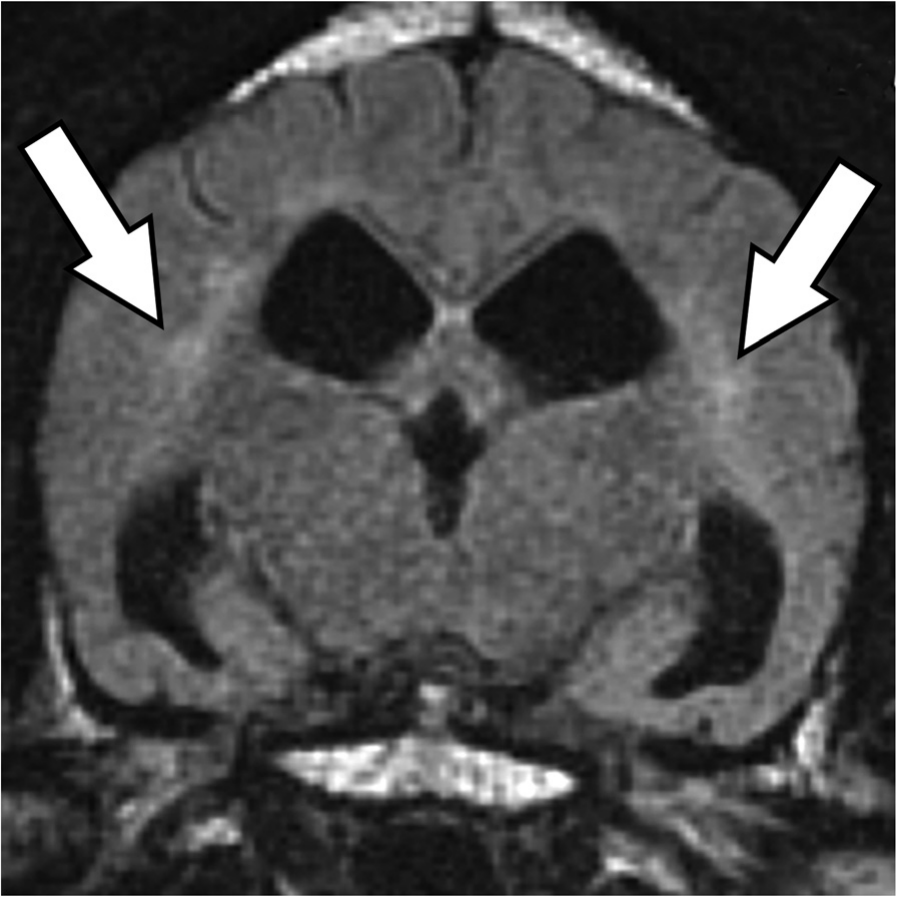
T2-W FLAIR sequence at the level of the interthalmic adhesion showing a hyperintense signal in the internal capsule peripheral to the lateral ventricles (arrows).

**Figure 3:**
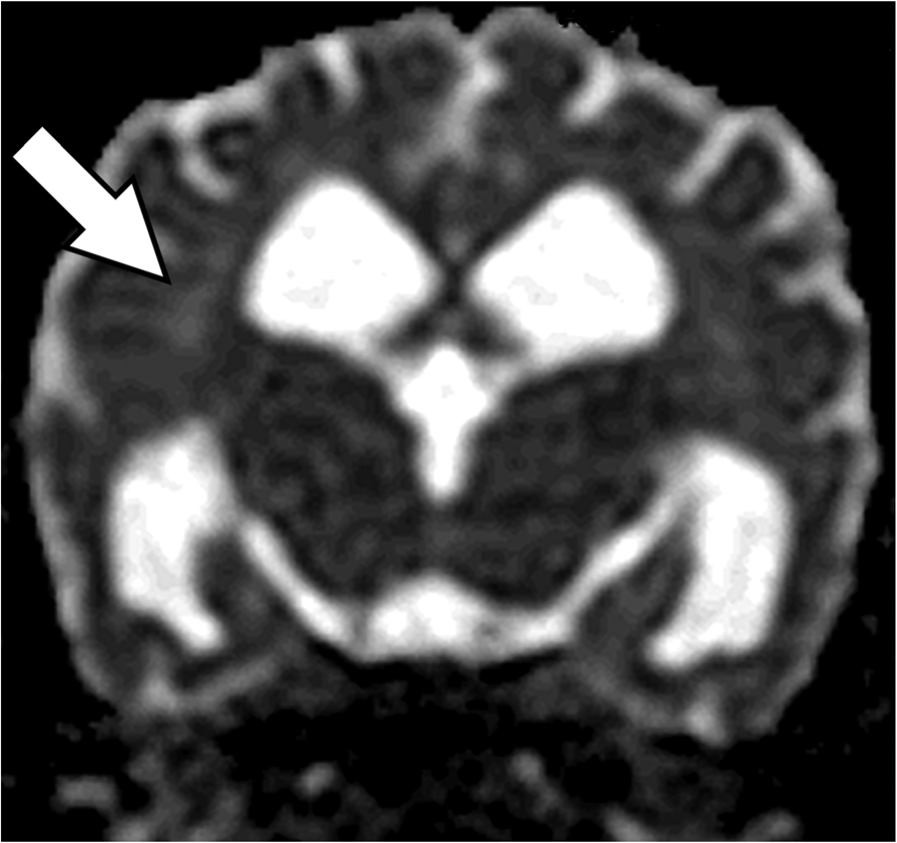
ADC Map at the level of the interthalmic adhesion showing a hyperintense signal in the internal capsule (arrow).

MR images showed a bilaterally symmetric T2-W and FLAIR hyperintense signal predominately affecting the white matter of the occipital and parietal lobes: tracking along the internal capsule and extending into the corona radiata peripheral to the lateral ventricles (Figures 1 and 2). The regions that were hyperintense on T2-W images were isointense on DWI and hyperintense on the ADC map (Figure 3). This was consistent with increased interstitial water content found in vasogenic oedema [15-17].

**Figure 4:**
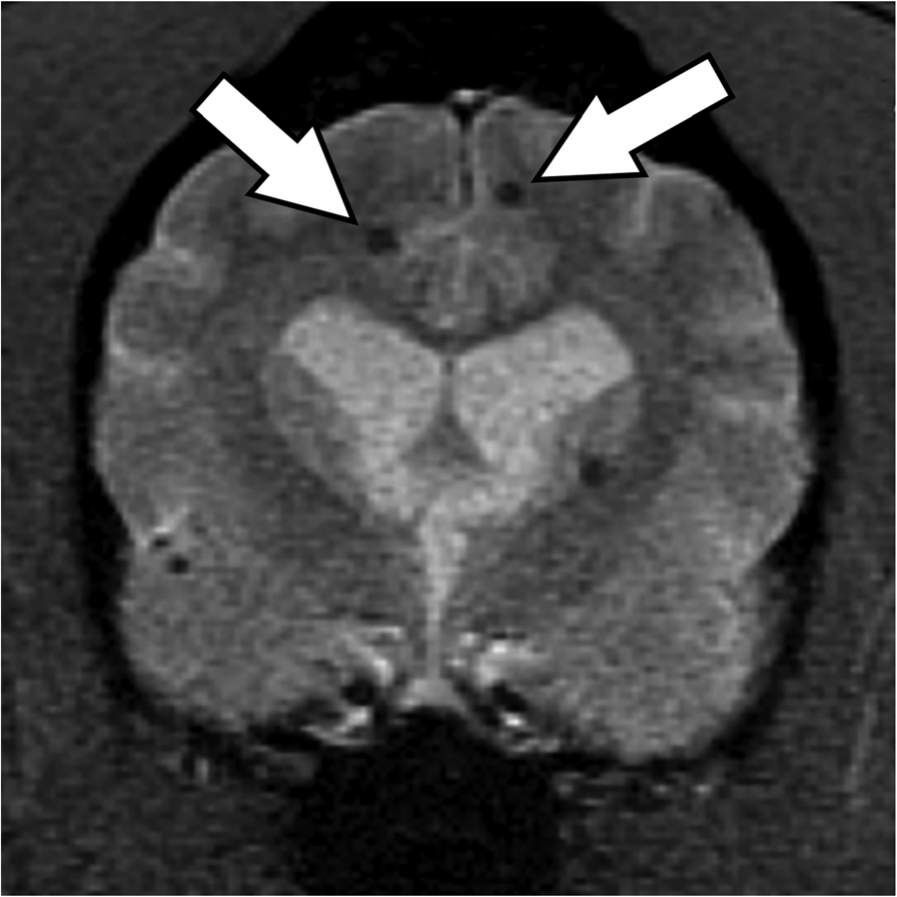
T2*-GRE at the level of the caudate nucleus showing 5 punctate hypointense lesions at the grey/white matter junctions (Arrows identify two lesions).

T2*-GRE images showed 32 small (<2.5 mm diameter) spherical lesions that were located in the cerebral hemispheres predominantly at grey/white matter junctions in the frontal, parietal, occipital and temporal lobes (Figure 4). Most lesions were only evident as signal voids on T2*-GRE with a small number of lesions being isointense to hypointense to white matter on T1-W, T2-W and FLAIR images. The lesions did not create a mass effect and there was no evidence of perilesional parenchymal changes. There was no contrast enhancement following gadolinium administration.

The dog was treated with the anti-hypertensive agent amlodipine besylate (Norvasc; Pfizer) at a dose of 2.5 mg once daily, and levetiracetam (Levitiracetam; generic) 250 mg every 8 hours. Myoclonic seizure activity responded within 12 hours to the administration of levetiracetam. The events decreased in frequency from every few minutes when in bright sunlight, to once or twice daily. There were no further episodes of altered mentation and collapse. Dietary alterations were recommended, and the dog was started on a commercial veterinary renal diet. At 9 months following the initial diagnosis, repeat examination of the dog showed infrequent myoclonic seizures. There were no further signs of acute ataxia, vocalizing and collapse, which were presumed to be secondary to hypertensive encephalopathy. The dog had stable renal parameters and was normotensive.

## Conclusions

Hypertensive encephalopathy (HE) is a syndrome of neurological abnormalities including seizure activity and altered mentation secondary to hypertension. In people, HE is considered part of PRES, and is characterized by vasogenic oedema that predominately affects the occipital and parietal lobes. Vasogenic oedema is thought to be secondary to a sudden elevation in blood pressure exceeding the autoregulatory capacity of the cerebral vasculature resulting in vessel wall injury and increased permeability of the blood–brain barrier. Altered blood–brain barrier permeability in conjunction with hyperperfusion allows the extravasation of fluid, macromolecules and red blood cells into the parenchyma [5-8,18-20]. Risk factors for the development of PRES in people include abrupt arterial hypertension, renal dysfunction, pre-eclampsia/eclampsia, immunosuppressive drugs, autoimmune disorders and systemic infection [5-7,18,19].

In this case, the distinguishing MRI feature was of a bilaterally symmetric T2-W and FLAIR hyperintense signal predominately affecting the subcortical white matter including the internal capsule and corona radiata of the occipital and parietal lobes. There was a lack of enhancement following intravenous gadolinium administration. DWI and ADC map changes were consistent with vasogenic oedema. Diffusion-weighted images are fundamental to the diagnosis of hypertensive encephalopathy, as it allows differentials such as ischaemia to be eliminated.

Hypertensive encephalopathy secondary to CKD has been described in the veterinary literature, although only one study reports MRI abnormalities consistent with hypertensive encephalopathy [9]. One animal in this study also had a single lesion with imaging characteristics suggestive of a cerebral microbleed. Pathologic findings in hypertensive encephalopathy are well documented in the veterinary literature. One study in dogs with CKD found that 3 of 14 (21%) dogs with averaged SAP values exceeding 180 mmHg experienced seizure activity [1]. Two of the three dogs had post mortem examination. One had findings consistent with cerebrovascular disease, while the other dog had vacuolization of myelinated fibre tracts, likely secondary to cerebral oedema. Another case series documented neurological abnormalities in 11 of 24 cats (46%) with hypertension secondary to diseases including CKD and hyperthyroidism. Two of these cats were subjected to necropsy and both had multifocal cerebral arteriosclerosis and focal regions of haemorrhage [21]. Cerebral oedema secondary to hypertension has been documented in cats secondary to reduced renal function or following renal transplantation [3,22]. Histopathologic findings include generalized white matter oedema with separation of the myelin sheaths by interstitial oedema [3].

Cerebrovascular disease, and in particular haemorrhage, may occur in conjunction with PRES with 32% of people in one study having microbleeds, hematomas or subarachnoid haemorrhage [7]. Cerebral microbleeds (CMB) in humans are secondary to amyloid angiopathy or from hypertension-induced vascular changes to small cerebral vessels [10-13]. As such they are considered to represent a vasculopathy with the leaking of read blood cells across the blood–brain barrier. Cerebral microbleeds can contribute to cognitive dysfunction, however the main significance of microbleeds in people is that they are considered to indicate a future risk of infarction [23,24]. As in this case, most CMBs in people are found distributed along the grey-white matter interface or in the superficial cortex [10]. On post-mortem examination, these focal areas adjacent to abnormal small vessels contain haemosiderin granules or haemosiderin containing macrophages/phagocytic microglia [10-13]. Cerebral microbleeds have been described in the veterinary literature although their significance and aetiology has not been established [14,25].

Differentials for MRI findings suggestive of hypertensive encephalopathy in this case would include a leukoencephalopathy secondary to toxins, myelin deficiency, leukodystrophy, or neurodegenerative diseases. The clinical course of these disorders has been described in the literature and is not consistent with this case. The owner, however, did not consent to further diagnostic tests including cerebrospinal fluid sampling meaning that they cannot be entirely excluded. The resolution of clinical signs with amlodipine and levetiracetam, however, would make them be considered much less likely. The dog in this case report underwent MR imaging immediately following frequent myoclonic activity. Post-ictal MRI changes include reversible unilateral or bilateral temporal and piriform lobe T2-weighted hyperintensities with varying contrast enhancement [26]. The distribution of white matter oedema in the parietal and temporal lobes in this case, was not considered to be characteristic of post-ictal MR imaging abnormalities.

The reflex myoclonus in this dog was presumed to be consistent with epilepsy: either primary, or secondary to cerebral microbleeds or a neurodegenerative disorder. The acute deterioration was considered to be secondary to hypertensive encephalopathy. Myoclonic seizures are rare in dogs, and have most commonly been described as a feature of Lafora disease and neuronal ceroid-lipofuscinosis [27-29]. The dog in this case report has been successfully medically managed with levetiracetam and amlodipine for one year following presentation making Lafora disease less likely. Lafora disease has also been reported in the veterinary literature to result in the dilation of the lateral ventricles secondary to cortical atrophy which was not a feature in this case [27]. Neuronal ceroid-lipofuscinosis also has a markedly different MR appearance with abnormalities consistent with cerebral and cerebellar atrophy [30,31]. Levetiracetam was selected as the anticonvulsive agent as it has been reported in people to be efficacious in the treatment of progressive myoclonus epilepsy [32].

This report describes the magnetic resonance appearance of hypertensive encephalopathy with concurrent cerebral microbleeds. Limitations of this report include the lack of a follow-up MRI to document resolution of the white matter T2-weighted hyperintensities with medical management. Future post-mortem analysis would also be useful. Hypertensive encephalopathy should be considered when evaluating animals that have an acute onset of neurological signs with concurrent systolic arterial hypertension.

## Abbreviations

CKD: Chronic kidney disease
SAP: Systolic arterial hypertension
PRES: Posterior reversible encephalopathy syndrome
MRI: Magnetic resonance imaging
T2*-GRE: T2*-weighted gradient recalled-echo sequences
RI: Reference interval
RL: Reference limit
IRIS: Internal Renal Interest Society
T1-W: T1-weighted
T2-W: T2-weighted
FLAIR: Fluid-attenuated inversion recovery
DWI: Diffusion-weighted images
ADC map: Apparent diffusion coefficient map
